# Functional characterisation of tumour suppressor PDCD4 reveals previously undisclosed role in the control of cell adhesion

**DOI:** 10.1093/nar/gkag071

**Published:** 2026-02-02

**Authors:** Veronica Dezi, Robert F Harvey, Tom Smith, Cameron H Cole, Mariavittoria Pizzinga, Mark Stoneley, Gavin D Garland, Emilie Horvilleur, Lajos Kalmar, Ritwick Sawarkar, Kathryn S Lilley, Anne E Willis

**Affiliations:** MRC Toxicology Unit, University of Cambridge, Tennis Court Rd, Cambridge CB2 1QRUK; MRC Toxicology Unit, University of Cambridge, Tennis Court Rd, Cambridge CB2 1QRUK; MRC Toxicology Unit, University of Cambridge, Tennis Court Rd, Cambridge CB2 1QRUK; MRC Toxicology Unit, University of Cambridge, Tennis Court Rd, Cambridge CB2 1QRUK; MRC Toxicology Unit, University of Cambridge, Tennis Court Rd, Cambridge CB2 1QRUK; Human Technopole, V.le Rita Levi-Montalcini, Milan 20157Italy; MRC Toxicology Unit, University of Cambridge, Tennis Court Rd, Cambridge CB2 1QRUK; MRC Toxicology Unit, University of Cambridge, Tennis Court Rd, Cambridge CB2 1QRUK; MRC Toxicology Unit, University of Cambridge, Tennis Court Rd, Cambridge CB2 1QRUK; MRC Toxicology Unit, University of Cambridge, Tennis Court Rd, Cambridge CB2 1QRUK; MRC Toxicology Unit, University of Cambridge, Tennis Court Rd, Cambridge CB2 1QRUK; Department of Biochemistry, University of Cambridge, Gleeson Building, Tennis Court Rd, Cambridge CB2 1QRUK; MRC Toxicology Unit, University of Cambridge, Tennis Court Rd, Cambridge CB2 1QRUK

## Abstract

PDCD4 is a multifunctional RNA-binding protein that has tumour suppressor function. To more fully understand how dysregulation of this protein contributes to carcinogenesis, we have carried out a comprehensive analysis of the role of PDCD4 in RNA metabolism in untransformed epithelial cells. We show that PDCD4 predominantly localises in the nucleus, where it interacts with proteins involved in a range of different RNA metabolic processes. We find that PDCD4 knockdown is associated with significant changes in either the expression or splicing of a number of transcripts, although it appears to have an indirect role in splicing. We identified the RNA targets of PDCD4 using iCLIP and observed an enrichment in binding to transcripts encoding cell adhesion and structural proteins. Consistent with these data, we show that PDCD4 acts as a general regulator of cell adhesion, which in a tumour setting would increase the metastatic potential of cells, and demonstrate that the nuclear localisation of PDCD4 is crucial in this process. Overall, the information obtained in untransformed cells provides a new perspective for the role of PDCD4 as a tumour suppressor.

## Graphical Abstract

**Graphical Abstract:**
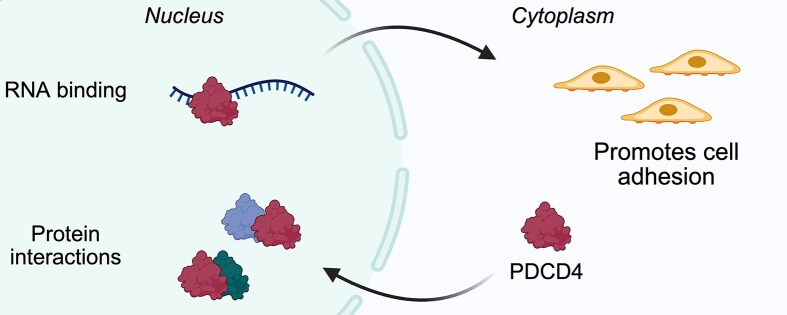

## Introduction

PDCD4 is a 64 kDa multifunctional nuclear-cytoplasmic shuttling protein which contains two MA3 domains and a disordered *N*-terminal region [1]. When localised in the cytoplasm, PDCD4 inhibits the activity of the eukaryotic initiation factor 4A (a DEAD-box RNA helicase and component of the eIF4F complex) via its MA3 domains by hindering its interaction with eIF4G [2–4]. EIF4A unwinds structured RNA elements in 5′ untranslated regions (UTRs) that would otherwise impede the scanning ribosome and since many mRNAs that encode proto-oncogenes and growth factors contain such highly structured 5′ UTRs, it has been proposed that through the inhibition of eIF4A activity, PDCD4 acts as a tumour suppressor [5, 6]. Accordingly, it has been shown that loss or down-regulation of PDCD4 is associated with tumorigenesis and poor patient outcome (reviewed in [7]).

In addition to its role as a translation initiation inhibitor, PDCD4 was recently found to stimulate translation termination *in vitro*. Interestingly, PDCD4 does not interact with known eukaryotic release factors (eRF), but the current model suggests that it has a dual role. First, PDCD4 disrupts the binding of eRF3 to PABP, hindering the loading of new release factors. Second, it stimulates the GTPase activity of eRF3 resulting in eRF3-GDP displacement and translation termination preventing subsequent termination rounds, and most likely, redirecting ribosomes to be inactivated in stress granules [8].

PDCD4 has also been reported to indirectly affect eukaryotic transcription. Thus, PDCD4 knockdown was found to enhance the expression of *SNAIL*, and so reduce *E-cadherin* levels, and induce the relocation of β-catenin to the nucleus, leading to the expression of urokinase receptor (*uPAR)* and *c-MYC* [9, 10]. The latter was shown to bind a specific region in the promoter of the mitogen-activated protein kinase (*MAP4K1*) enhancing its expression [10]. This ultimately affects the activity of AP-1, a JUN/JUN homo- or JUN/FOS heterodimeric complex upstream of genes involved in cell proliferation, transformation, invasion, and angiogenesis [11]. Additionally, PDCD4 was also reported to alter transcription via transcription factor sequestration; in PC3 cells, the interaction of PDCD4 with TWIST1 results in inhibition of transcription of *YB-1* [12], whereas in macrophages, LPS-induced degradation of PDCD4 promotes *IL-10* production through the release of TWIST2 [13].

The expression of PDCD4 is regulated post-transcriptionally by microRNAs such as *miR-21* [14–16], and this process is intricately regulated by the RBPs HuR, TIA1 and La [17–19] to maintain tight control of PDCD4 levels. Post-translational regulation of PDCD4 occurs via mTOR signalling, culminating in the phosphorylation of PDCD4 on residues Ser67 and Ser76 by protein kinase B (AKT) and p70 ribosomal S6 kinase (p70S6K) [20]. PDCD4 also contains two putative nuclear localisation signals (NLSs) in positions 58–64 and 241–250 and its nuclear translocation is dictated by phosphorylation on Ser457 by either AKT or ribosomal S6 kinase (RSK) [21, 22]. Notably, nuclear PDCD4 expression is associated with a good prognosis in Luminal A and Luminal B-like breast cancers [23].

To date, the majority of studies on PDCD4 have focused on its role as a translation suppressor and the impact of its downregulation in cancer. However, RNA-binding proteins (RBPs) such as PDCD4 are multifunctional and play key roles in maintaining cellular homeostasis [24, 25]. Therefore, it was essential to gain a greater understanding of the role of PDCD4 in cells derived from healthy tissues to more fully understand its dysregulation in carcinogenesis.

Here, we identify the protein partners of PDCD4 in untransformed cells, where it primarily resides in the nucleus, and demonstrate that these proteins are involved in various RNA metabolic processes. In agreement with these data, PDCD4 knockdown is associated with significant changes in the expression and/or splicing patterns of numerous transcripts. Using improved individual nucleotide resolution CLIP (iCLIP), we found that PDCD4 shows enriched binding to transcripts encoding cell adhesion and structural proteins. Consistent with these findings, PDCD4 knockdown or overexpression of mutant PDCD4 that resides only in the cytoplasm affects cell adhesion and spreading. These data provide new insights into the role of PDCD4 as a tumour suppressor.

## Material and methods

### Cell culture and live-cell imaging

MCF10A cells were cultured at 37°C with 5% CO_2_ in Dulbecco’s modified Eagle’s medium (DMEM)/F12 (1:1) supplemented with horse serum (5%), recombinant human epidermal growth factor (20 ng/ml, Peprotech), recombinant human insulin (10 µg/ml, Sigma−Aldrich), hydrocortisone (500 ng/ml, Sigma−Aldrich), and cholera toxin (10 ng/ml, Sigma−Aldrich).

MDA-MB231 cells were cultured at 37°C with 5% CO_2_ in DMEM, high glucose, pyruvate, GlutaMAX™ Supplement, supplemented with 10% Fetal bovine serum (FBS).

### Cell treatments

MCF10A cells were starved for either 4 h or overnight with Dulbecco’s modified Eagle’s medium (DMEM)/F12 (1:1) supplemented with horse serum (0.1%). Starved cells were stimulated with recombinant human insulin (10 µg/ml, Sigma-Aldrich) for 30 min according to the experimental design.

Before collecting MCF10A cells for experiments, the growth media were refreshed 4 h in advance.

To block CRM1-dependent, NES-dependent nucleo-cytoplasmic translocation, cells were treated with 20 nM Leptomycin B (LMB) (Sigma-Aldrich) for 4 h along with media replacement.

### Transfection

MCF10A cells were reverse-transfected with 10 nM siRNA (for human PDCD4: ON-TARGETplus L-004438–00-0005, Dharmacon) using Lipofectamine 2000 reagent (Thermo Fisher Scientific) according to manufacturers’ instructions for either 48 or 72 h.

MDA-MB-231 cells were transfected using with 0.5 ug pcDNA3.1-WT-Flag PDCD4 or pcDNA3.1-S457A-Flag PDCD4 using Lipofectamine 3000 reagent (Thermo Fisher Scientific) according to manufacturers’ instructions. All constructs were generated and cloned by GeneArt Custom Gene Synthesis (Thermo Fisher Scientific).

### Immunofluorescence (IF)

Cells were seeded onto glass coverslips and treated according to the experimental design. Cells were then fixed and permeabilised with cold 100% methanol. Primary antibody incubation was performed at 4°C overnight with the indicated antibodies (Supplementary Table 5). Secondary fluorescent antibodies were incubated for 1 h at room temperature (Supplementary Table 5). Cell nuclei were stained with 1:1000 Hoechst 33 258 (Thermo Fisher Scientific) for 15 min at room temperature. Images were acquired on Zeiss LSM 880 confocal (63 × oil objective) and analysed with ZEISS ZEN Microscope Software.

### Image analysis

Immuno-fluorescence images were analysed using a custom-made Python script. First, cell segmentation masks were generated using CellPose [26]. From the cell segmentation masks, membrane-only masks were generated using a boundary-detection algorithm from scikit-image [27], specifically find_boundaries. Then, we applied binary dilation (binary_dilation, with parameters mode=”thick” and thickness = 5) to obtain an area of fixed thickness around the cell membrane. The masks thus generated were then used to measure membrane and whole cell mean intensities for each image. Mean membrane intensity was normalised by whole cell intensity. Statistical significance was assessed using Welch’s *t*-test.

### Cell adhesion assay

The xCELLigence real-time cell analysis (RTCA) platform was used with modifications specific to the application of cell adhesion assays [28]. Briefly, cells were seeded onto a six-well plate, and knockdown experiments were carried out as described above. Trypsin-EDTA was used to detach MCF10A or MDA-MB-231 cells and then inactivated with either 10% HS or 10% FBS. Following washes with PBS, 17 000 cells/well were seeded onto a RTCA E-Plate previously coated with 20 µg/ml collagen from calf skin (Merck). 0.1% BSA coated wells were used as a negative control. Cell adhesion was assessed over a period of 3 h with measurements every 2 min.

### Cell lysis and protein quantification

Cells were washed with cold PBS and lysed in RIPA buffer (50 mM Tris HCl pH 7.5, 150 mM NaCl, 1% Triton X-100, 0.1% SDS, 0.5% sodium deoxycholate) supplemented with cOmplete mini EDTA free protease inhibitor cocktail (Roche) and 1X PhosSTOP phosphatase inhibitor cocktail (Roche). Protein concentration was quantified using PierceTM BCA Protein Assay Kit (Thermo Fisher Scientific) and cell lysates were diluted in SDS loading buffer (50 mM Tris (pH 6.8), 2% SDS, 10% glycerol, and 0.1% bromophenol blue), supplemented with 50 mM DTT and heated at 95°C for 5 min.

### Co-Immunoprecipitation (co-IP) and analysis

Around 1.5 mg Dynabeads® M-270 Epoxy (Thermo Fisher Scientific) were coupled with 5 µg of PDCD4 polyclonal antibody (Supplementary Table 5) overnight according to manufacturers’ instructions. MCF10A cells from a 15 cm^2^ dish were washed in cold PBS and lysed in fresh lysis buffer (20 mM Tris-HCl pH 8.0, 137 mM NaCl, 0.3% NP-40, 2 mM MgCl2), supplemented with 1X cOmplete mini EDTA free protease inhibitor cocktail (Roche). Cell lysate was left rotating for 10 min at 4°C and passed through a 23-gauge needle 10 times before being quantified using PierceTM BCA Protein Assay Kit (Thermo Fisher Scientific). 1.5 mg of protein was incubated with the antibody-Dynabeads mix at 4°C for 2 h with gentle rotation and then washed five times with Lysis Buffer before elution. For Western blot analysis, beads were resuspended in SDS-loading buffer, incubated at 70°C for 10 min (shaking at 700 rpm) and supplemented with DTT (100 mM final concentration). Samples were heated at 95°C for 5 min. For Mass Spectrometry analysis, beads were resuspended in 100 µl 0.5% Rapigest in 25 mM AMBIC solution, incubated at 80°C for 10 min, shaking at 700 rpm and the supernatant collected for further analysis. Protein quantification was performed using the Pierce 660 nm assay (Pierce™). Around 5 µg of total proteins were reduced with 4 mM DTT (Thermo Scientific™) at 60°C for 10 min, alkylated with 14 mM IAA (final concentration; # I3750, Sigma–Aldrich) for 30 min at room temperature in the dark before DTT quenching. Trypsin digestion (200 ng sequencing-grade trypsin solution, Promega Corporation) was performed at 37°C for 16 h with constant shaking (500 rpm). Peptides were eluted and cleaned by strong cation exchange (SCX) (Thermo Scientific™), followed by drying in a speed vacuum and storing at −20°C until use.

For LC-MS/MS acquisition, peptides were resuspended in 20 µL of 0.1% TFA containing 3% acetonitrile. Peptides were analysed using a Vanquish Neo UHPLC system (Thermo Scientific) coupled to an Exploris 480 mass spectrometer (Thermo Scientific) equipped with FAIMS Pro for gas phase separation. The peptide samples were loaded onto a trapping column (Thermo Scientific, PepMap100, C18, 300 µm x 5 mm) using partial loop injection for 3 min at a flow rate of 15 µL/min with 0.1% (v/v) FA in 3% acetonitrile. The sample was resolved on an analytical column (Easy Spray C18, 75 µm x 750 mm, 2 µm) at a flow rate of 300 nL/min using a gradient from 97% A (0.1% formic acid) to 3% B (80% acetonitrile, 0.1% formic acid) over 50.4 min, followed by an increase to 45% B over an additional 8 min.

LC-MS/MS data were acquired using two FAIMS CVs (50V and 70V), with each FAIMS experiment having a maximum cycle time of 1.5 s. For both FAIMS experiments, the data-dependent acquisition (DIA) program consisted of a full scan MS at a resolution of 120 000 (AGC set to 300% (3e6 ions) with a maximum fill time of 50 ms). MS/MS was performed at a resolution of 15 000 (AGC set to 200% (4e5 ions) with a maximum fill time of 22 ms) with an isolation window of 1.2 m/z and an HCD normalised collision energy of 26. To avoid repeated selection of peptides for MS/MS, the program used a 40-s dynamic exclusion window.

Raw data were imported and processed in Proteome Discoverer v3 (Thermo Fisher Scientific). The raw files underwent iterative database searches using Proteome Discoverer with SequestHF and Inferys rescoring algorithms against the *Homo sapiens* database [29] (updated on 20 220 524), which contains human protein sequences from UniProt/Swiss-Prot. Common contaminant proteins (various types of human keratins, BSA, and porcine trypsin) were included in the database. Spectra identification was performed using the following parameters: MS accuracy set to 10 ppm, MS/MS accuracy of 0.02 Da for spectra acquired in the Orbitrap analyser, allowance for up to two missed cleavage sites, carbamidomethylation of cysteine as a fixed modification, and oxidation of methionine as a variable modification. The Percolator node was utilised for false discovery rate estimation, and only rank 1 peptide identifications with high confidence (FDR < 1%) were accepted.

Label-free quantification was conducted based on the precursor ion area of identified peptides.

Peptide-level output from Proteome Discoverer was parsed and further filtered using the parse_features function in camprotR (version 0.0.0.9000) [30]. Along with metadata, peptide abundances were stored in an MSnSet object, “diff.median’ normalised and aggregated to protein level abundances using the MSnbase R package (version 2.24.2) [31].

Proteins identified only in the PDCD4 co-IP replicates were referred to as class I; proteins identified in two out of three PDCD4 co-IP replicates but in none of the IgG samples, were referred to as class II proteins in the paper. With the rest of identified proteins, missing values were imputed using either the “MinProb” or the “knn” methods in MSnbase. Missing values in the PDCD4 co-IP were imputed only if present in one replicate; whilst up to two missing values per protein were allowed to be imputed for the IgG co-IP. The rationale was to be more stringent in accepting possible PDCD4-binding proteins while trying to identify as many contaminants as possible in the IgG. All identified proteins except for those in class I and class II were subjected to limma (version 3.54.2) testing [32].

### Western blot analysis

Protein samples were separated on 4–12% SDS PAGE gel (Thermo Fisher Scientific), wet transferred on to PVDF membrane (Bio-Rad), blocked in 5% milk and detected with the indicated antibodies (Supplementary Table 5). IRDye Secondary Antibodies (LI-COR) were used in conjunction with an Odyssey Imager to detect proteins and bands were quantified with Image Studio™ Lite Quantification Software.

### Polysome profiling

Cells were seeded in a 15 cm^2^ dish and treated according to the experimental design. On the day of the experiment, the media was refreshed 4 h prior to harvesting. Cells were washed twice with 1X PBS- cycloheximide (100 μg/ml) and lysed in low salt gradient lysis buffer (20 mM Hepes pH 7.0, 10 mM NaCl, 5 mM MgCl2, 0.2 M sucrose, 0.5% NP-40, 5 μl RNasin, and 0.1 mg/ml cycloheximide). After 3-min incubation on ice, cells debris were pelleted at 1.300 g for 5 min at 4°C. The supernatant was quantified using PierceTM BCA Protein Assay Kit (Thermo Fisher Scientific).

Cell lysates were layered on sucrose gradients (10–50%) and centrifuged at 38 000 rpm for 2 h at 4°C. Gradient fractions were collected at 1-min intervals and the absorbance measured at 254 nm using a UA-6 UV/Vis detector (Teledyne Isco).

### RNA extraction and RT-qPCR

RNA extraction was performed from polysomal fractions with TRIzol™ LS Reagent according to the manufacturer’s instructions. Reverse transcription was carried out with Superscript™ IV (Thermo Fisher Scientific) and random hexamers (Invitrogen), according to the manufacturer’s instructions. The qPCR was performed using iTaq Universal SYBR® Green Supermix (Bio-Rad) with specific primers (Supplementary Table 5) using the Quantstudio 6 Flex real-time PCR system (Thermo Fisher Scientific).

### Orthogonal organic phase separation (OOPS)

MCF10A (one 10 cm^2^ plate) or MDA-MB-231 (one 6 cm^2^ plate) cells were washed in cold PBS and cross-linked with UVC (400 mJ/cm^2^) irradiation prior to lysis in TRIzol™ Reagent (Thermo Fischer Scientific). RBPs were isolated according to the protocol described in Queiroz *et al.* [33, 34] with the addition of a second RNA-digestion step using RNase1 for 30′ at 37°C. Methanol-precipitated proteins were quantified using the BCA assay. Around 10 µg total protein was used for Western blot analysis.

### Improved individual nucleotide resolution CLIP protocol (iiCLIP) and data analysis

Cells from a 15 cm^2^ dish were washed in cold PBS and cross-linked with UVC (150 mJ/cm^2^) irradiation. iiCLIP was performed as in F.C.Y Lee et. al. [35] using 1 mg of lysate and 5 µg PDCD4 antibody (Proteintech).

The final multiplexed library was sent for single-end sequencing using Illumina NextSeq500 High Output 75-cycle sequencing (Illumina Inc.).

The quality of Fastq files was checked with the FastQC tool (Andrews, S. (2010). FastQC: A Quality Control Tool for High Throughput Sequence Data [Online]. Available online at: http://www.bioinformatics.babraham.ac.uk/projects/fastqc/).

The iCLIP analysis was performed using iCount, a Python module and associated command-line interface (CLI), which provides all the commands needed to process protein-RNA iCLIP interaction data. The reference genome for mapping was downloaded from Ensembl (release 88). Prior to the peaks function in iCount, cross-linked sites present in the IgG replicates were subtracted from the PDCD4 replicates and the latter merged in a single bed file using the group function, and further filtered to keep only peaks with a score greater than 5. Heatmaps for intron-exon and exon-intron junctions were generated using the rnamaps function in iCount.

iCount output files were eventually further analysed in RStudio; differential binding analysis was performed with Deseq2 (version 1.38.3), plots generated with ggplot2 (version 3.4.4) and Gene ontology (GO) enrichment analyses performed using goseq (version 1.50.0) taking the normalised read counts as bias factor.

The genomic coordinates of introns were extracted from TxDb.Hsapiens.UCSC.hg38.knownGene and the overlap with PDCD4 peaks coordinates was determined using the findOverlap function in the GenomicRanges R package (version 1.50.2). Because multiple peaks could potentially map to the same intron, the unique number of introns bound by PDCD4 was defined and the score of single peaks mapping to each intron summed up. A control dataset comprising random introns was generated to account for potential biases by sampling randomly from all introns, with the probability of selection weighted by the length of the intron.

ANOVA testing was implemented to provide insights into the significance of differences in lengths among PDCD4-bound, IgG-bound and control introns.

### RNA sequencing (RNA-seq) and data analysis

RNA was extracted with the RNeasy Mini Kit (QIAGEN), and RNA integrity was assessed using the Agilent 4150 TapeStation (Agilent). Libraries were prepared according to TruSeq Stranded Total RNA Library Prep Human/Mouse/Rat (Illumina Inc.) system. Paired-end sequencing was performed using Illumina NextSeq500 High Output 150-cycle (2 × 75 bp) sequencing (Illumina Inc.). Demultiplexed files were downloaded from BaseSpace (Illumina Inc.), and lanes concatenated into a single fastq per sample. Reads were then pseudo-aligned to the human transcriptome reference GRCh38 (release 109) using Salmon, and transcript-level estimates were imported in R studio using the R package tximport (version 1.26.1). Differential gene expression (DGE) and differential transcript expression (DTE) were carried out using the tximport function and DESeq2 (version 1.38.3).

In order to test if the effect of PDCD4 knockdown on the transcriptome (in either direction) was linked to the binding of the protein to those affected RNAs, we tested whether there was an association between the genes with differential binding (iCLIP) and differential abundance (RNA-Seq). Since higher RNA abundance is expected to lead to higher power to detect differential binding or abundance, we generated a null distribution for the expected number of genes affected in both binding and abundance using a random sampling procedure which accounted for this bias. Genes binned based on their abundance using the read counts in the iCLIP and RNA-Seq datasets. Within each bin, the number of significant genes in either iCLIP or RNA-Seq analysis was identified, and the same number of genes was randomly selected from each bin. The overlap between the randomly selected genes from the iCLIP and RNA-Seq datasets was determined, and this procedure was repeated ‘n’ times to obtain a null distribution for how many genes would be both differential bound and differentially abundant by chance. An empirical *P*-value was then calculated by comparing the observed overlaps to the null distribution.

Alternative splicing analysis was performed using rMATS (version 4.1.2) and it was run with the -t paired –readLength 75 parameters and the gencode.v44.annotation.gtf annotation. Only Junction Counts files were considered for further analysis using the R package maser (version 1.16.0). Alternative splicing events were defined as statistically significant if they have an FDR ≤ 5%, an absolute inclusion level difference > 0.1, and inclusion read counts + skipped read counts ≥ 10.

To test if there was a link between changes in skipped exons (SE) and the binding of PDCD4 near the AS event, a new genomic range was created spanning from the 3′ end of the upstream exon to the 5′ end of the downstream exon. Then the overlap with the PDCD4 peak file was determined using the findOverlap function in the GenomicRanges R package (version 1.50.2) and a metric reflecting the absolute effect size and statistical significance was calculated as: abs(IncLevelDifference)*-log10(*P-*Value). To analyse the relationship between this metric and PDCD4 binding around those exons, a logistic regression generalised linear model was fitted.

### Additional statistical analysis

Additional statistical analysis was performed using the unpaired t-test, assuming normal distribution, or one way ANOVA when comparing two or more groups, respectively using Graphpad Prism version 10 (Graphpad software, San Diego, USA). Unless otherwise stated, error bars represent mean ± standard deviation of N replicates.

## Results

### PDCD4 interacts with additional RNA-binding proteins

**Figure 1.**
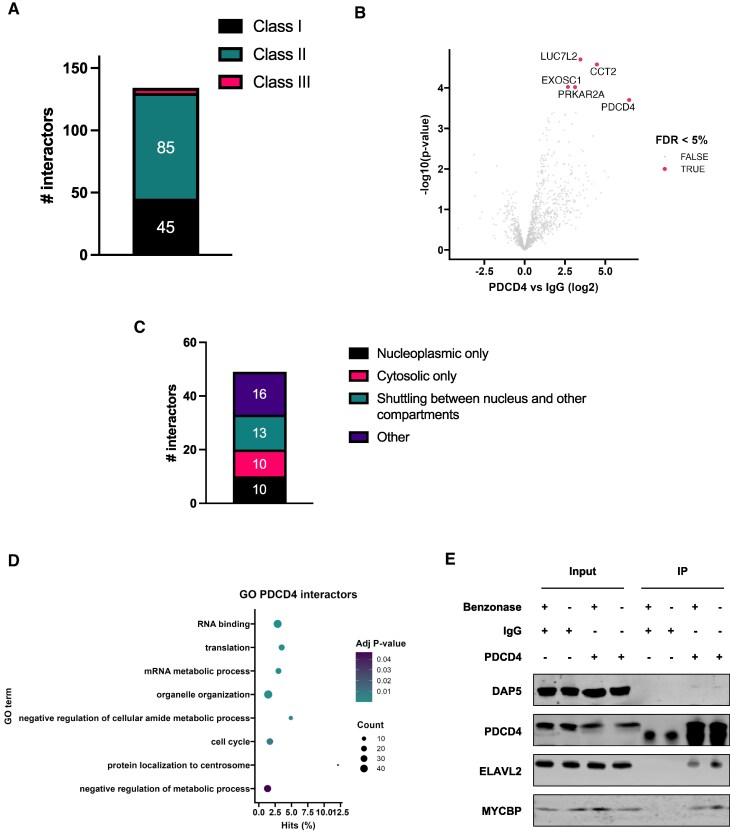
PDCD4-bound proteome. **(A)** Stacked bar chart showing the number of proteins immunoprecipitated with PDCD4 identified in three biological replicates (class I), two biological replicates (class II), or identified as enriched following Linear Models for Microarray and RNA-Seq Data (LIMMA) analysis (class III). **(B)** Volcano plot displaying the enrichment of PDCD4 interactors following limma analysis; *x*- and *y*-axes represent log2 fold-change differences and statistical significance (as the negative log of *P*-values), respectively (Adj *P*-value < 0.05). **(C)** Stacked bar chart reporting the number of protein interactors of classes I and III localising to the selected subcellular niches according to human protein atlas. **(D)** Top molecular function GO terms over-represented in proteins enriched in the PDCD4 co-IP; *P*-values were calculated taking into account protein abundance (from Paxdb) and adjusted according to Benjamini–Hochberg (see Materials and methods). **(E)** Representative Western blot showing input and co-IP samples in the presence of absence of Benzonase (250U, 1 h). Input samples were taken after lysate was separated for PDCD4 and IgG immunoprecipitation.

To gain a better understanding of the role of PDCD4 in cells derived from healthy tissues, the binding partners of PDCD4 were identified using co-immunoprecipitation (co-IP) and label-free quantification mass spectrometry (MS). PDCD4 primarily resides in the nucleoplasm of unstressed cells [36] (Supplementary Fig. 1A); however, as it rapidly diffuses to the cytoplasm upon cell lysis (Supplementary Fig. 1B), we were able to utilise milder buffer conditions to extract PDCD4 and the interacting proteins. In total, compared to the control IgG samples, 45 proteins interacted with PDCD4 in all three biological replicates (class I), 85 in two biological replicates (class II) and 4 were identified as enriched following Linear Models for Microarray and RNA-Seq Data (limma) analysis (FDR 5%, class III) [32] (Fig. 1A, B and Supplementary Table 1). As PDCD4 is predominantly a nucleoplasmic protein (Supplementary Fig. 1A), it was unsurprising that two previously reported cytoplasmic interactors, PABP and eIF4A [2, 3, 37], were not amongst the targets identified in the MS analysis, although PABP was slightly enriched against the IgG control (Supplementary Fig. 1C). Furthermore, consistent with the nuclear localisation of PDCD4, out of the 49 protein partners (45 class I and 4 class III), 23 were either exclusively nucleoplasmic or nuclear-cytoplasmic shuttling proteins, whereas only 10 were reported to be cytoplasmic, as annotated by the Human Protein Atlas (https://www.proteinatlas.org/) (Fig. 1C). The nuclear/cytoplasmic ratio was even higher when including proteins from class II (Supplementary Fig. 1D).

Gene Ontology (GO) analysis identified more than 40 proteins functionally annotated as “RNA binding” as well as significant enrichment for “translation” and “mRNA metabolic process” (Fig. 1D, Supplementary Table 1). Western blot analysis was performed to validate the interactions, including the two most enriched proteins from class I, ELAV-like protein 2 (ELAVL2, also known as HELN1 or HuB) and MYC Binding Protein (MYCBP), and one from class II, eukaryotic translation initiation factor 4 gamma 2 (eIF4G2, also known as DAP5) (Fig. 1E). To determine whether these interactions with PDCD4 were direct or RNA-dependent, immunoprecipitation was carried out in the presence or absence of the nuclease Benzonase. RNA digestion had no impact on DAP5 binding, indicating that this was a direct interaction with PDCD4 (Fig. 1E). The amount of DAP5 recovered in a PDCD4 co-IP is modest, which is likely due to these proteins residing in different subcellular compartments. Conversely, the binding of ELAVL2 and MYCBP was reduced after Benzonase treatment, suggesting a partly nucleic acid-mediated interaction (Fig. 1E).

Taken together, these data suggest that within the nucleus of cells derived from healthy tissues, PDCD4 interacts with a substantial number of RNA-binding proteins, most of which are either localised within the nucleus or are nuclear/cytoplasmic shuttling proteins.

### PDCD4 interacts with RNA in MCF10A cells

PDCD4 is considered to be an RBP, however only a few targets have been described so far [38, 39]; therefore, it was important to assess the genome-wide PDCD4-bound transcriptome.

The RBPbase database (v0.2.1 alpha) integrates high-throughput RBP detection studies in different organisms and cell types. According to this database, PDCD4 was classified as a candidate RBP in studies that also identified the well-characterised RBPs HuR, HRNPC1/2, and PTBP1 (Supplementary Fig. 2A, Supplementary Table 2). However, PDCD4 was not identified in many other studies, which could reflect the low expression of the tumour suppressor protein in the cell lines employed.

**Figure 2.**
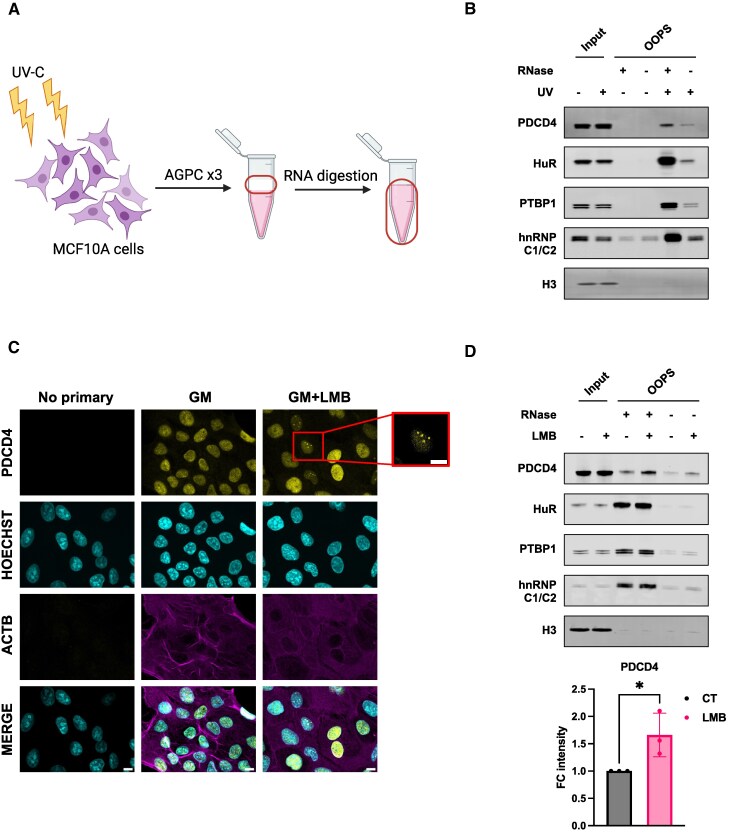
PDCD4 binds RNA in MCF10A cells. **(A)** Schematic representation of OOPS. **(B)** Western Blotting analysis of input and OOPS samples in either UV-C irradiated or unirradiated MCF10A cells, in the presence or absence of RNAse (1, A and T1) treatment. Blots shown are representative of three independent biological experiments. **(C)** Confocal microscopy of either growing (GM) or LMB-treated MCF10A (20 nM final concentration, 4 h); Cells were stained against ACTB, PDCD4 and cells nuclei with Hoechst (scale bar, 10 μm). Images were acquired on Zeiss LS880 confocal microscope. **(D)** Top, western blotting analysis of input and OOPS samples following LMB treatment (20 nM final concentration, 4 h) and UV-C irradiation. Blots shown are representative of three independent biological experiments. Bottom, PDCD4 densitometry measured from the RNase treated lanes with the Image Studio Li-COR software and shown as barplot with statistics (Unpaired *t*-test was applied, *N* = 3, * = *P* value < 0.05).

To test whether PDCD4 could bind RNA in cells derived from normal breast epithelium, MCF10A cells were either exposed to UVC (400 mJ/cm^2^) irradiation or left unirradiated and the RNA-binding proteome was enriched using OOPS [33, 34] (Fig. 2A). RBPs enriched at the aqueous-organic interface due to UVC cross-linking were released by RNA digestion and recovered for western analysis. In a similar manner to the canonical RBPs HuR, PTBP1, and hnRNP C1/C2, PDCD4 was enriched at the interface after UV cross-linking and was released from it after RNA digestion, demonstrating that PDCD4 has RNA binding capacity (Fig. 2B).

Due to its nucleoplasmic localisation and robust interaction with nuclear proteins, we next investigated whether the RNA binding of PDCD4 was primarily in the nucleus of MCF10A cells, rather than originating from the small fraction of the protein in the cytoplasm. Since PDCD4 rapidly diffuses from the nucleus upon cell lysis (Supplementary Fig. 1B), it was not possible to perform OOPS on fractionated cell lysates. Therefore, the growth media (GM) of MCF10A cells was supplemented with Leptomycin B (LMB), a potent and specific inhibitor of XPO1-mediated nuclear export, to ensure PDCD4 nuclear retention. Interestingly, while LMB prevented PDCD4 export from the nucleus of growing cells (Fig. 2C and Supplementary Fig. 2B) and even prevented export in response to serum starvation (Supplementary Fig. 2C), it also led to a noticeable re-localisation of the protein to small nuclear foci (Fig. 2C). OOPS was subsequently performed on LMB-treated cells which revealed an increase in RNA binding exclusively for PDCD4, without any similar effect observed for other tested RNA-binding proteins (Fig. 2D).

Collectively, these data indicate that PDCD4 binds directly to RNA and the majority of these interactions occur in the nucleus of MCF10A cells.

### PDCD4 binds to coding sequences and introns, but it does not directly affect splicing

**Figure 3.**
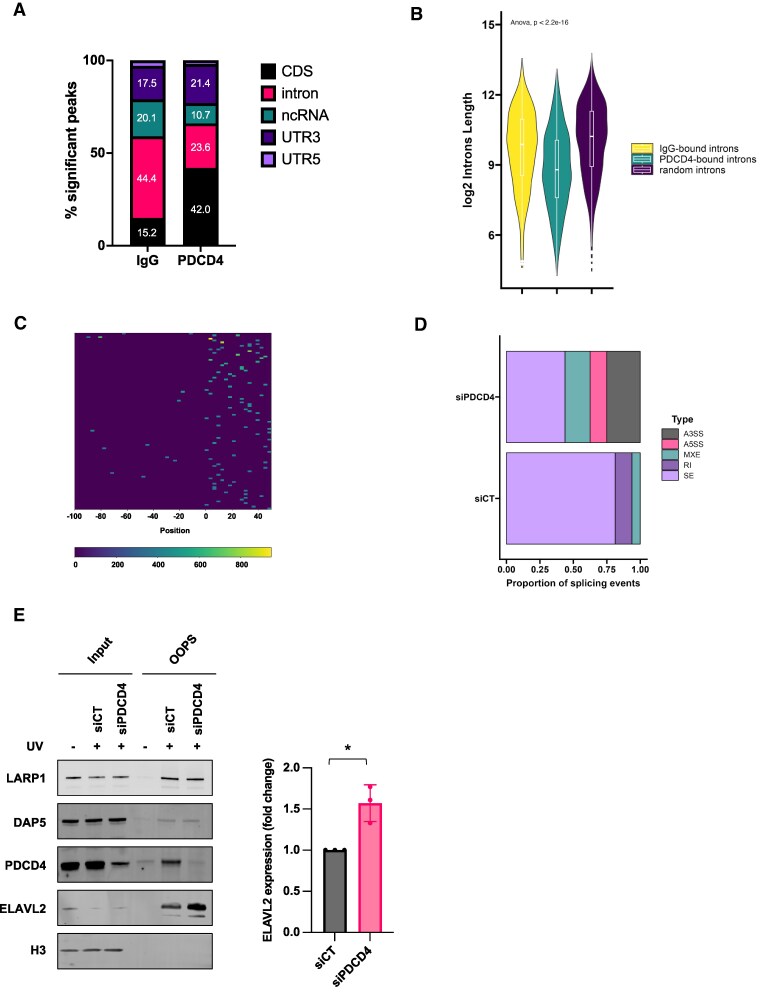
PDCD4 binding preferences. **(A)** Stacked bar chart representing the RNA biotypes enriched in either IgG or PDCD4 iCLIP datasets (FDR 5%, score > 5). **(B)** Violin plot reporting the length (expressed in logarithm scale) of the introns bound by either PDCD4 or IgG as well as the length of randomly sampled and weighted introns. Statistical comparisons were performed using ANOVA. **(C)** Heatmap highlighting the distribution of PDCD4 peaks relative to intron-exon junctions, expressed as count per million (CPM). D) Stacked bar chart reporting the proportion of significant splicing events (FDR < 5% and -0.1 < deltaPSI > 0.1) in either siCT or siPDCD4 cells; each alternative splicing (AS) type is represented by a colour. Type of splicing events shown are skipped exon (SE), mutual exclusive exon (MXE), retained intron (RI), alternative 5′-end splice site (A5SS), alternative 3′-end splice site (A3SS). **(E)** Left, western Blotting analysis showing input and OOPS samples in either siCT or siPDCD4 MCF10A cells (± UV-C irradiation). Blots shown are representative of three independent biological experiments. Right, bar chart reporting the densitometry of ELAVL2 measured with the Image Studio Li-COR software (Unpaired *t*-test was applied, *N* = 3, * = *P* value < 0.05).

To identify RNA transcripts directly targeted by PDCD4 and expand our current knowledge on the function of the tumour suppressor, an improved individual-nucleotide resolution UV cross-linking and immunoprecipitation (iCLIP) assay [35] was performed and the PDCD4-bound molecules were sequenced with next-generation sequencing (NGS) on the Illumina platform. Principal component analysis (PCA) showed a pronounced transcriptome divergence between isotype control (IgG) and PDCD4-iCLIP (Supplementary Fig. 3A). When focusing on highly significant peaks (score greater than 5 and FDR < 5%), 42% mapped to coding sequences (CDS) consistent with studies on individual mRNAs [38], 23.6% mapped to intronic regions, 21.4% to the 3′UTR, 10.7% and 2.3% to noncoding regions and to the 5′UTR, respectively (Fig. 3A). The majority of intronic peaks belong to pre-mRNAs rather than to long noncoding RNAs (lncRNAs) (Supplementary Fig. 3B). The binding of PDCD4 to intronic regions, although unexpected, is consistent with its nuclear localisation under growing conditions. To rule out the possibility that the RNA binding of PDCD4 within introns was simply a reflection of the size of these genomic elements, the length of the PDCD4-bound introns was compared to those bound by the IgG and a random weighted control. One-way ANOVA statistical testing showed a significant difference in the lengths of the introns among the tested groups with the PDCD4-bound introns shorter than those in both the random weighted and the IgG controls (Fig. 3B). A small percentage of significant peaks mapped to intron-exon junctions (Fig. 3C) with the highest peaks in Plectin (PLEC), Solute Carrier Family 7 Member 5 (SLC7A5, also known as LAT1), Mucin 1 (MUC1), and Keratins 81 (KRT81) and 7 (KRT7). However, the only GO term enriched for peaks mapping to intron-exon junctions was “RNA-binding” (Supplementary Fig. 3C, Supplementary Table 3). Interestingly, almost no enrichment towards the 5′ Splice Site (SS) was observed, suggesting a specificity for the 3′SS region (Supplementary Fig. 3D).

Binding of PDCD4 to introns and in proximity of the 3′SS could potentially suggest a role in RNA splicing. Therefore, PDCD4 expression was reduced by siRNA, RNA-seq carried out, and this dataset used for a replicate multivariate analysis of transcript splicing (rMATS). Compared to controls cells, PDCD4 silenced MCF10A cells showed an impact on 28 alternative splicing events, primarily skipped exons (SE, *n* = 20), such as in CD44, the CDC42 binding protein kinase beta (CDC42BPB), and GRB10 interacting GYF protein 2 (GIGYF2) (Fig. 3D, Supplementary Fig. 3E and Supplementary Table 4). Furthermore, intron retention (RI, *n* = 2), alternative 3′ splice site (A3SS; *n* = 4), and alternative 5′ splice site (A5SS; *n* = 2) events were also altered (Fig. 3D and Supplementary Table 4). To identify whether PDCD4 had a direct or indirect role in splicing, for each splicing event (SE) detected in rMATS, the absolute effect size (delta percent spliced in) was multiplied by the statistical unlikeliness (-log10(*P*-value)) to obtain a metric summarising the change in splicing. Logistic regression was applied to determine whether changes in splicing were associated with PDCD4 binding in close proximity to the skipped exon. According to this statistical model, there was no significant association between PDCD4 binding and exon skipping (Supplementary Fig. 3F).

As PDCD4 was found to interact with multiple RBPs, including splicing-associated proteins (Fig. 1D and Supplementary Table 1), we then asked whether the RNA-binding capacity of these interactors could be affected by PDCD4 knockdown. OOPS was performed following PDCD4 knockdown by siRNA, and we observed a significant increase in ELAVL2 binding to RNA (Fig. 3E).

These data show that PDCD4 binds primarily to the coding sequences of target RNAs. Despite the observed interaction with shorter introns, as well as a preference for binding to the 3′SS region, binding of PDCD4 to RNA does not appear to directly regulate RNA splicing. However, these data suggest the interaction of PDCD4 with other RBPs may impact their RNA binding and subsequent RNA processing.

### PDCD4 binds to mRNAs encoding for cell adhesion molecules and structural proteins

**Figure 4.**
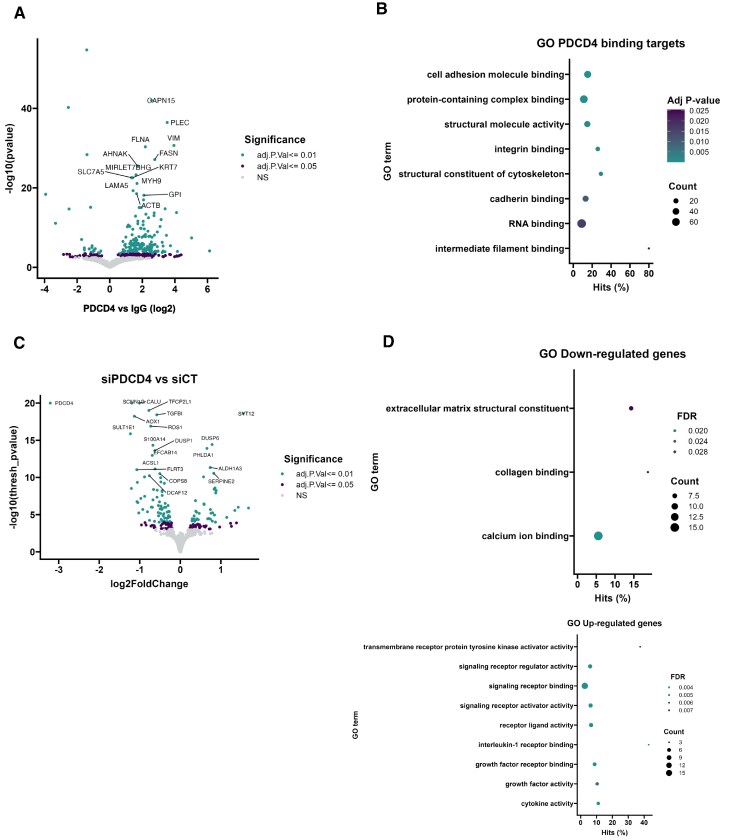
PDCD4 binds to cell adhesion molecules. **(A)** Volcano plot representing the enrichment of PDCD4-bound RNAs compared to the IgG control; *x* and *y* axes represent log2 fold change and –log10 *P*-value, respectively (adjusted *P*-value (BH adjustment) less than 0.05 and 0.01, respectively). **(B)** Top eight molecular function GO terms over-represented in RNAs enriched in the PDCD4 iCLIP; *P*-values were calculated taking into account read counts and adjusted according to Benjamini–Hochberg (see Materials and methods). **(C)** Volcano plot representing differential gene expression upon PDCD4 silencing; *x* and *y* axes represent log2 fold change and –log10 *P*-value, respectively; in green and red are transcripts with adjusted *P*-value (BH adjustment) less than 0.05 and 0.01, respectively. D) Molecular function GO terms over-represented in genes significantly down-regulated (on the top) or up-regulated (on the bottom) upon PDCD4 silencing; *P*-values were calculated taking into account gene length and read counts and adjusted according to Benjamini–Hochberg (see Materials and methods).

Analysis of our PDCD4 iCLIP dataset revealed specific binding to a variety of mRNA transcripts (Fig. 4A) and GO term analysis highlighted an enrichment for transcripts encoding cell adhesion molecules and structural proteins, including cytoskeletal components (Fig. 4A and B). To determine how PDCD4 may regulate gene expression, we carried out RNAseq analysis following its siRNA-mediated depletion. We identified 113 genes and 274 transcripts that were downregulated (FDR < 5%, Fig. 4C, Supplementary Fig. 4A, 4B and Supplementary Table 4) upon PDCD4 knockdown with a small, yet significant, enrichment in the term “extracellular matrix structural constituent” (Fig. 4D). In total, 25 transcripts found to be significantly (FDR < 5%) enriched in the PDCD4 iCLIP dataset were also differentially expressed upon gene knockdown (Supplementary Fig. 4B, C and Supplementary Table 4), suggesting that PDCD4 may directly or indirectly regulate the stability of these transcripts. Taken together these data suggest that PDCD4 interacts with several transcripts encoding cell adhesion and structural molecules, and it regulates the expression of different genes, in some cases, through direct molecular interactions with their respective mRNAs.

### Knockdown of PDCD4 is associated with changes in cell adhesion

**Figure 5.**
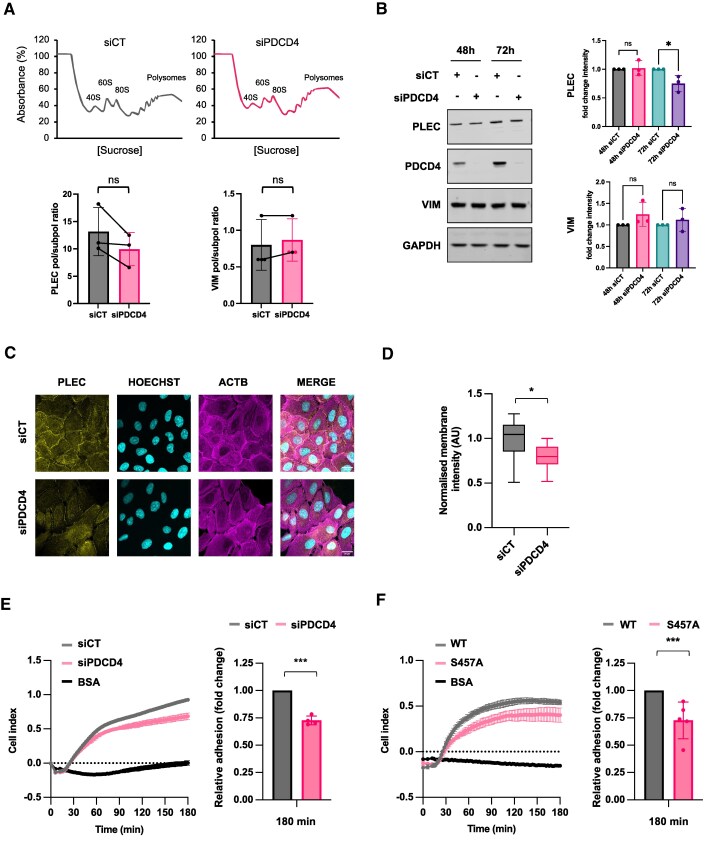
PDCD4 in cell adhesion. **(A)** Top, representative polysome profiling traces for siCT and siPDCD4 MCF10A cells. Bottom, bar charts representing the loading of both PLEC (left) and VIM (right) on either subpolysomal or polysomal fractions, expressed as a ratio (Unpaired *t*-test was applied, *N* = 3, ns = not significant). **(B)** Left, western blotting of siCT and siPDCD4 MCF10A cells upon either 48- or 72-h knockdown. Right, bar chart reporting the densitometry of either PLEC (top) or VIM (bottom) measured with the Image Studio Li-COR software (Unpaired *t*-test was applied, *N* = 3, *= *P*-value ≤ 0.05). **(C)** Confocal microscopy of MCF10A cells transfected with siCT and siPDCD4 for 48 h. Cells were stained against PLEC and ACTB, and cells nuclei with Hoechst (scale bar, 20 μm). Images were acquired on Zeiss LS880 confocal microscope. **(D)** Box plot showing quantification of PLEC protein at the cell membrane following PDCD4 knockdown in MCF10A cells. Mean PLEC intensity at the membrane was normalised to mean intensity of the whole cell. Quantification was obtained from 10 (siCT) or 11 (siPDCD4) fields of view across three independent biological replicates (Welch’s *t*-test was applied, siCt = 173 cells, siPDCD4 = 171 cells, *= *P*-value ≤ 0.05). **(E)** Left, a representative line plot showing the adhesion of MCF10A cells transfected with siCT or siPDCD4 for 48 h, using the RTCA xCELLigence platform over 180 min. Cell index is an arbitrary value that can be used to infer cell attachment. Right, bar chart showing the relative difference of adhesion in siPDCD4 compared to siCT cells after 180 min (unpaired *t*-test was applied, *N* = 4 biological replicates, *** = *P*-value < 0.001). **(F)** Left, a representative line plot showing the adhesion following overexpression of WT or S457A PDCD4 in MDA-MB-231 cells using the RTCA xCELLigence platform over 180 min. Cell index is an arbitrary value that can be used to infer cell attachment. Right, bar chart showing the relative difference of adhesion in S457A PDCD4 compared to WT cells after 180 min (unpaired *t*-test was applied, *N* = 5 biological replicates, *** = *P*-value < 0.001).

To validate our iCLIP data we assessed two of the most enriched transcripts which encoded plectin (PLEC) and vimentin (VIM) (Supplementary Fig. 5A). Importantly, RNAseq analysis revealed no significant differences in the levels of both mRNAs after PDCD4 depletion (Supplementary Fig. 5B). To determine whether the binding of PDCD4 may affect the translation of *PLEC* and *VIM* transcripts, we carried out polysome profiling and RT-qPCR analysis following PDCD4 siRNA knockdown (Fig. 5A). These data demonstrate that although *VIM* mRNA did not show any difference in the association with polysomes when compared to control cells, we overserved a trend in the reduction of *PLEC* mRNA from polysomes after 48 h of PDCD4 depletion. The subtle drop in *PLEC* mRNA association with heavy polysomes resulted in a small, yet significant, reduction in PLEC protein levels after 72 h of PDCD4 knockdown (Fig. 5B). Moreover, the cellular localisation of PLEC was altered after 48-h PDCD4 depletion, showing decreased accumulation at cell membranes (Fig. 5C and D). Taken together, these correlative data suggest that PDCD4 may be required for the efficient translation of *PLEC* mRNA.

In epithelial cells, both cell adhesion molecules and structural components of the cytoskeleton are essential to ensure adequate adhesion to the extracellular matrix (ECM). As the most enriched GO terms within our PDCD4 iCLIP data were related to cell adhesion, we determined the impact of PDCD4 on cell adhesion using the RTCA xCELLigence platform [28], which measures changes in electrical impedance to infer cell attachment in a label-free manner over 180 min. Compared to control cells, PDCD4 depleted MCF10A cells adhered to the ECM more slowly, confirming a role for PDCD4 in this process (Fig. 5E). Importantly, the effect on MCF10A cell adhesion was observed within 48 h of PDCD4 knockdown, whereas PLEC protein did not decrease until 72 h of knockdown (Fig. 5B), suggesting that the reduction in PLEC was unlikely to be solely responsible for the effect on cell adhesion.

PDCD4 is a tumour suppressor protein and we hypothesise, given the potential role in cell adhesion, that PDCD4 loss could be associated with increased metastases and therefore worse disease prognosis. Unsurprisingly, the overall probability of survival in breast cancer patients with lower PDCD4 expression is decreased compared to patients with higher PDCD4 levels (Supplementary Fig. 5C).

Our previous data suggests that PDCD4 predominantly binds to its target transcripts in the nucleus (Fig. 2). To confirm that the nuclear interaction between PDCD4 and transcripts controlling cell adhesion was not limited to MCF10A cells, we used the triple-negative breast cancer (TNBC) epithelial cell line, MDA-MB-231. Compared to MCF10A cells, MDA-MB-231 cells express low levels of PDCD4 (Supplementary Fig. 5D). We therefore utilised transient expression of either WT or mutant S457A PDCD4, which cannot translocate to the nucleus [21] (Supplementary Fig. 5E). In support of our MCF10A siPDCD4 data, cells expressing mutant S457A PDCD4 adhere more slowly compared to cells overexpressing wild-type PDCD4 (Fig. 5F), suggesting that the nuclear function of PDCD4 is crucial to the regulation of transcripts promoting cell adhesion. Moreover, whereas overexpression of WT PDCD4 increased PLEC protein expression, PLEC was significantly lower in control and S457A PDCD4 overexpressing cells (Supplementary Fig. 5F). In support of our data from LMB treated MCF10A cells (Fig. 2D), S457A PDCD4 expressed in MDA-MB-231 cells showed reduced RNA binding (Supplementary Fig. 5G), correlating with a reduction of PLEC protein levels (Supplementary Fig. 5F). Taken together, these data demonstrate that the nuclear function of PDCD4 is crucial to the regulation of PDCD4-bound mRNAs.

## Discussion

There have been numerous studies on the function of PDCD4 as a tumour suppressor protein in cancer-derived cells, however, very little research has been carried out to understand the role of PDCD4 in non-tumorigenic tissues. Therefore, a greater understanding of the role of this protein in cells derived from control tissues was warranted. We show that in untransformed epithelial MCF10A cells, PDCD4 predominantly localises in the nucleus, where it interacts with many RNA-binding proteins which are either similarly nuclear-based or nuclear/cytoplasmic shuttling proteins (Fig. 1C and Supplementary Fig. 1D). Given this localisation of PDCD4, it was therefore unsurprising that two well-described interactors in tumour-derived cells, PABP and eIF4A1, were not identified as significant binding targets in MCF10A cells, and only a small proportion of eIF4G2 (also known as DAP5) was found to interact with PDCD4 (Fig. 1E). We confirmed that PDCD4 binds to RNA and that some of the interactions occur in the nucleus (Fig. 2). Consistent with these data, using iCLIP we identify that while PDCD4 binds predominately to CDSs, it also interacts with non-coding regions (Fig. 3A and Supplementary Fig. 3B). We find no evidence for a direct role of PDCD4 in splicing; therefore, we hypothesise that the interaction of PDCD4 with intronic regions suggests with an indirect role in splicing or alternatively that these regions act as a “bait” for nuclear retention of PDCD4 under control conditions.

Interestingly, a significant number of PDCD4 RNA targets identified in our iCLIP analysis encode for proteins involved in cell adhesion and/or structure, including PLEC (Fig. 4A and B). This finding aligns with our data showing that PDCD4 knockdown or overexpression of mutant PDCD4 that cannot shuttle to the nucleus, affects cell adhesion and spreading (Fig. 5E and F). Interestingly, we observed a reduction in cell adhesion within 48 h PDCD4 depletion (Fig. 5E), whereas PLEC protein only decreased from 72 h (Fig. 5B), suggesting the dysregulation of PLEC alone may not be responsible for reduced cell adhesion. However, we did observe mislocalisation of PLEC protein following PDCD4 depletion from 48 h (Fig. 5C and D). Although the mechanism of PLEC mislocalisation after PDCD4 depletion is not currently clear, it may be a direct consequence of cells being less adherent or, if PDCD4 directly regulates processes that determine PLEC localisation, it is possible that the delocalisation of PLEC may contribute to the observed effect on cell adhesion. In addition, the lack of any physical interaction between PDCD4 and eIF4A in MCF10A cells, as well as our observations with the phospho-mutant in MDA-MB-231 cells, suggest that cell adhesion is unlikely to be regulated via PDCD4-mediated inhibition of eIF4A. The impact of PDCD4 on cell adhesion is also consistent with data showing PDCD4 overexpression impairs the migration of hepatocellular carcinoma (HCC), ovarian, and endometrial cancer cells [40–42], suggesting that PDCD4 may be a general regulator of adhesion. Furthermore, analysis also shows that high expression of PDCD4 is associated with better prognosis of breast cancer patients (Supplementary Fig. 5C).

**Figure 6.**
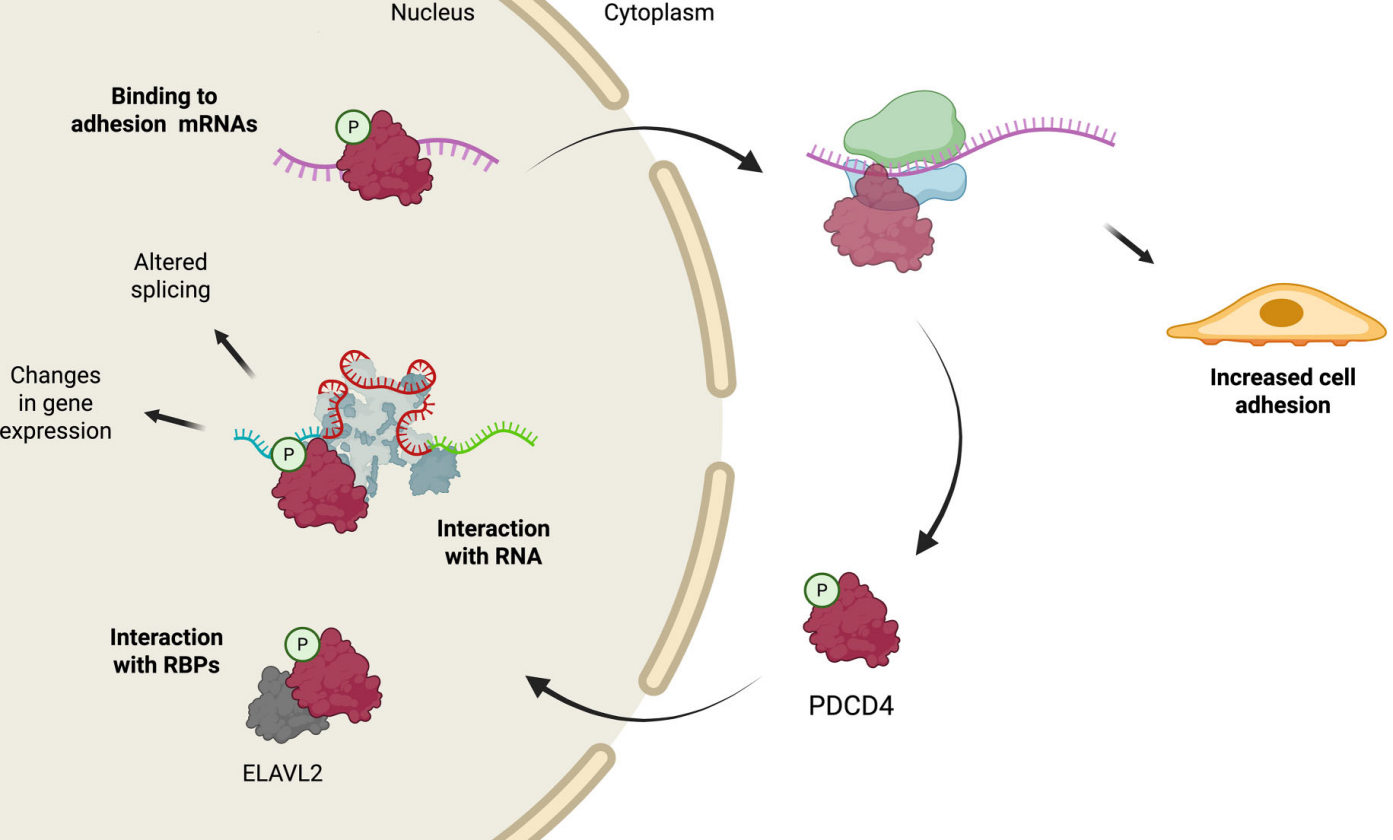
Proposed model of PDCD4 regulation of cell adhesion. Under growing conditions, PDCD4 is phosphorylated on Ser457 by AKT or ribosomal S6 kinase (RSK) and migrates to the nucleus. Here, PDCD4 interacts with multiple RBPs, including ELAVL2, ultimately affecting RNA levels and splicing regulation. Nuclear PDCD4 also interacts with different RNA molecules, some of which encode for cell adhesion-related factors, increasing cell attachment to ECM. Created in BioRender. Harvey, R. (2025) https://BioRender.com/sc1f4kh.

RBPs do not function individually, instead they form complexes composed of various subsets of RBPs under different cellular conditions that work together to coordinate a wide array of cellular processes [24, 25]. PDCD4 is similar in this regard and our data show that it displays localisation-dependent interactions with RBPs, with a predominantly nuclear localisation in untransformed cells. We propose that PDCD4 interacts with protein partners and target RNAs in the nucleus of untransformed cells. These PDCD4-containing RNP complexes are subsequently exported to the cytoplasm where they associate with the ribosomal machinery, and this ultimately regulates cell adhesion (Fig. 6). These data, obtained from studying the function of PDCD4 in untransformed cells, expand our understanding of the role of PDCD4 as a tumour suppressor and provide an additional explanation for poor cancer prognosis in patients with low PDCD4 expression.

## Supplementary Material

gkag071_Supplemental_Files

## Data Availability

Mass spectrometry data have been deposited in ProteomeXchange with the primary accession code PXD054188. All the sequencing data generated in this paper have been deposited in the Gene Expression Omnibus under accession numbers GSE272497 and GSE272498. Any additional information required to reanalyse the data reported in this paper is available from the corresponding contact upon request.
