# Supplementary material for: Functional characterisation of tumour suppressor PDCD4 reveals previously undisclosed role in the control of cell adhesion
Source: Nucleic Acids Res. 2026 Feb 2;54(3):gkag071. doi: 10.1093/nar/gkag071 (PMC12862386; doi:10.1093/nar/gkag071)
Supplement: gkag071_Supplemental_Files [file gkag071_supplemental_files.zip › PDCDC4_Supfig_Revised.pdf]

SUPPLEMENTARY TABLES

Supplementary Table 1. PDCD4 co-IP

Supplementary Table 2. PDCD4 in RBPbase database

Supplementary Table 3. PDCD4 iCLIP

Supplementary Table 4. siPDCD4 KD DGE, DTE and splicing

Supplementary Table 5. Antibodies and primers used in this study

Suppl. Figure 1

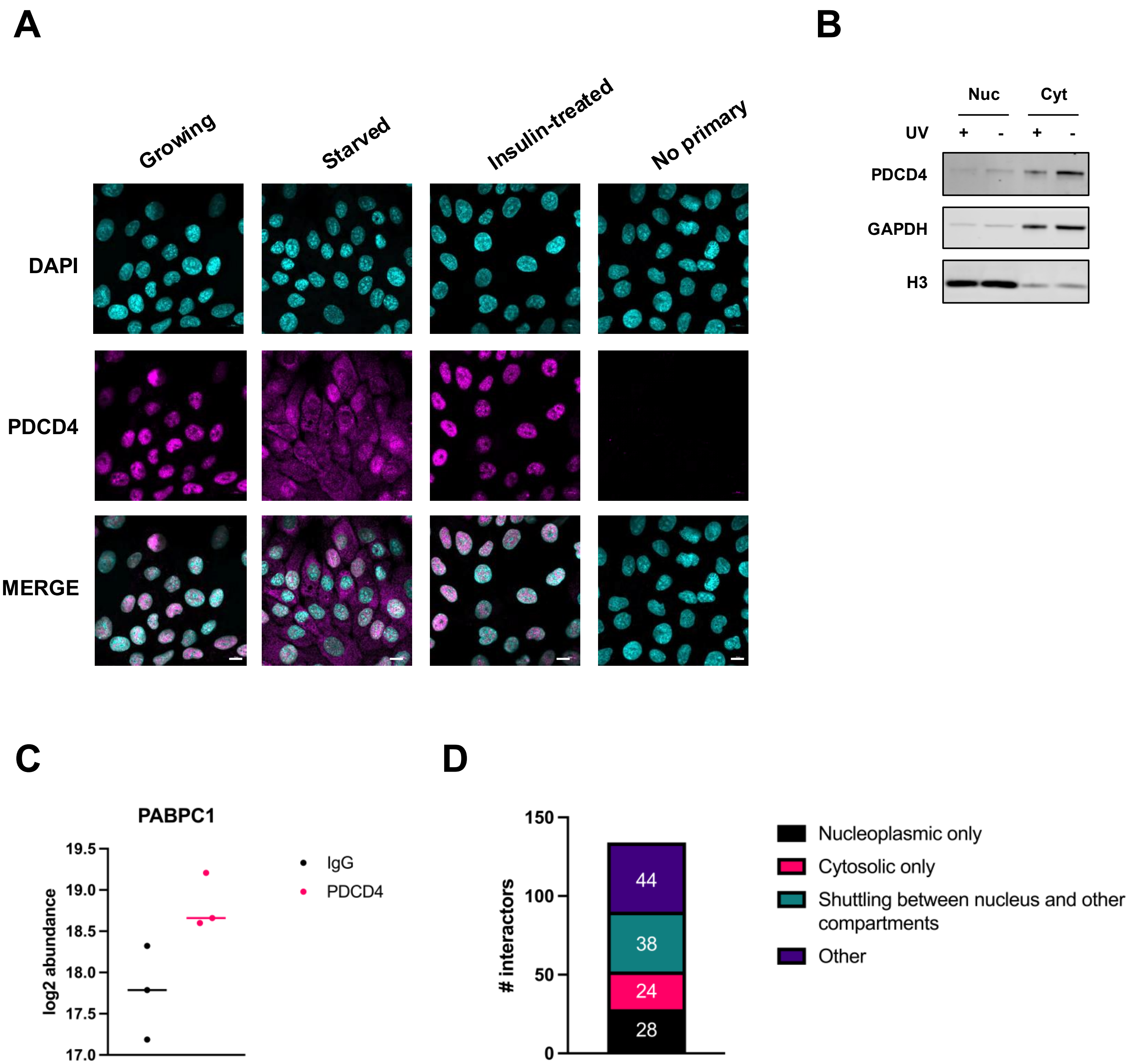

Supplementary Figure 1. A) Confocal microscopy showing PDCD4 localisation in MCF10A cells under either growing conditions, 4-hour starved or 30’ insulin-treated (1.7 μM final concentration). Cells nuclei were stained with Hoechst; scale bar, 10 μm. Images were acquired on Zeiss LS880 confocal microscope B) Western blot for PDCD4 following subcellular fractionation, +/- UV-C irradiation. C) Scatter plot showing the log2 abundance of PABPC1 in either the IgG or PDCD4 co-IP-MS replicates. D) Stacked bar chart reporting the number of protein interactors of classes I, II and III localising to the selected subcellular niches according to Human Protein Atlas.

Suppl. Figure 2

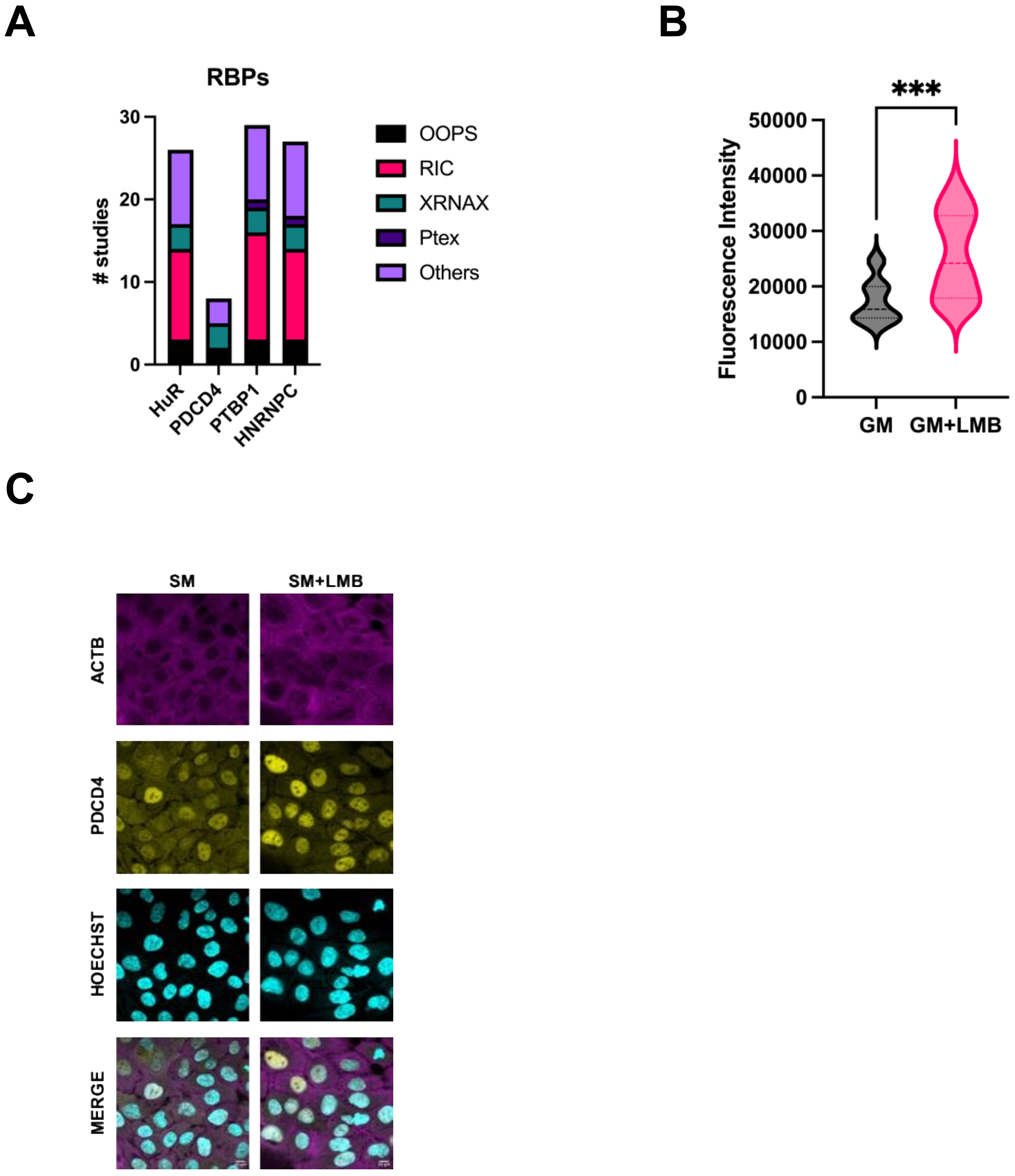

Supplementary Figure 2. A) Stacked bar chart displaying the number of studies in which the selected RBPs have been considered RNA-binding candidates. B) Violin plot showing the distribution of nuclear fluorescence intensities for PDCD4 in individual cells grown in either GM or GM+LMB (in parallel to Figure 2C). Nuclei were segmented using Hoechst staining to generate nuclear masks and mean nuclear PDCD4 intensity was quantified for each cell. An unpaired two-tailed t-test was used to assess significance (*** = p-value <0.001). C) Confocal microscopy of serum starved (SM) or starved and LMB-treated MCF10A cells (20 nM). Cells were stained against ACTB (magenta), PDCD4 (yellow) and cells nuclei with Hoechst (scale bar, 10 µm). Images were acquired on Zeiss LS880 confocal microscope (N=1).

Suppl. Figure 3

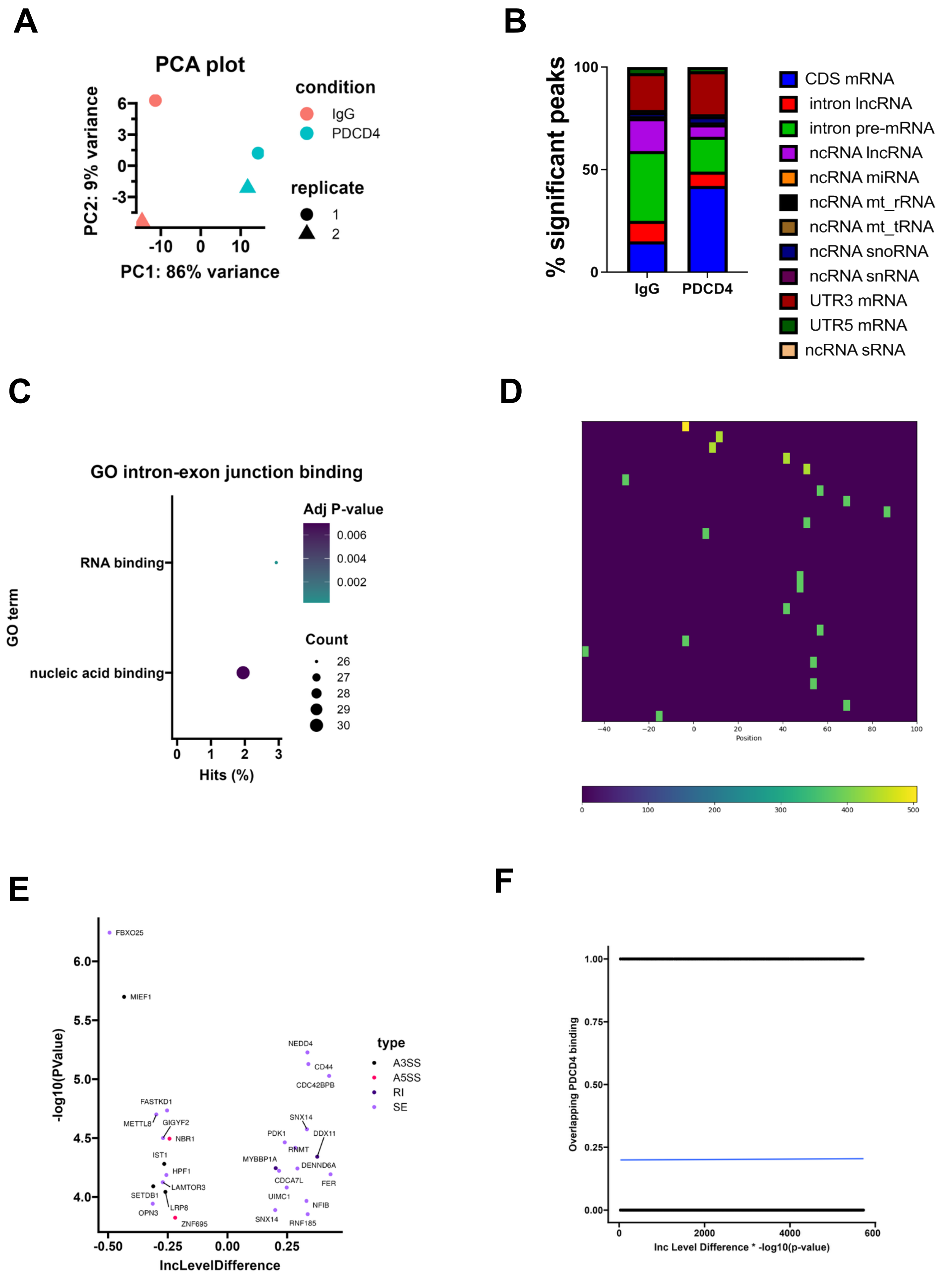

Supplementary Figure 3. A) Principal component analysis (PCA) of PDCD4 and IgG iCLIP samples. B) Stacked bar chart representing the RNA subtypes enriched in either IgG or PDCD4 iCLIP datasets (FDR 5%, score> 5). C) Significant MF GO terms over-represented in the list of PDCD4 peaks mapping to the intron-exon junctions; P-values were calculated taking into account gene length and read counts and adjusted according to Benjamini–Hochberg (see Methods). D) Heatmap highlighting the distribution of PDCD4 peaks relative to exon-intron junctions, expressed as count per million (CPM). E) Simplified volcano plot showing only significant (FRD< 5% and -0.1< deltaPSI >0.1 and sum read count >10, See methods) splicing events; x and y axes represent inclusion level difference (deltaPSI) and –log10 P-value, respectively; events with negative deltaPSI represent have higher inclusion in the siPDCD4 cells; different AS types are shown in different colours. F) Scatterplot depicting the relationship between the splicing effect size and the likelihood of PDCD4 binding around skipped exon (SE) events. The x-axis represents the rank of the metric derived from multiplying the inclusion level difference (IncLevelDifference) by the negative logarithm of the p-value (-log10(PValue)), providing a measure of absolute effect size and statistical significance. The y-axis indicates the presence (1) or absence (0) of PDCD4 binding overlaps near the SE events. Data points represent individual SE events, with a fitted binomial logistic regression model (blue line) showing the predicted probability of PDCD4 binding as a function of the ranked effect metric.

Suppl. Figure 4

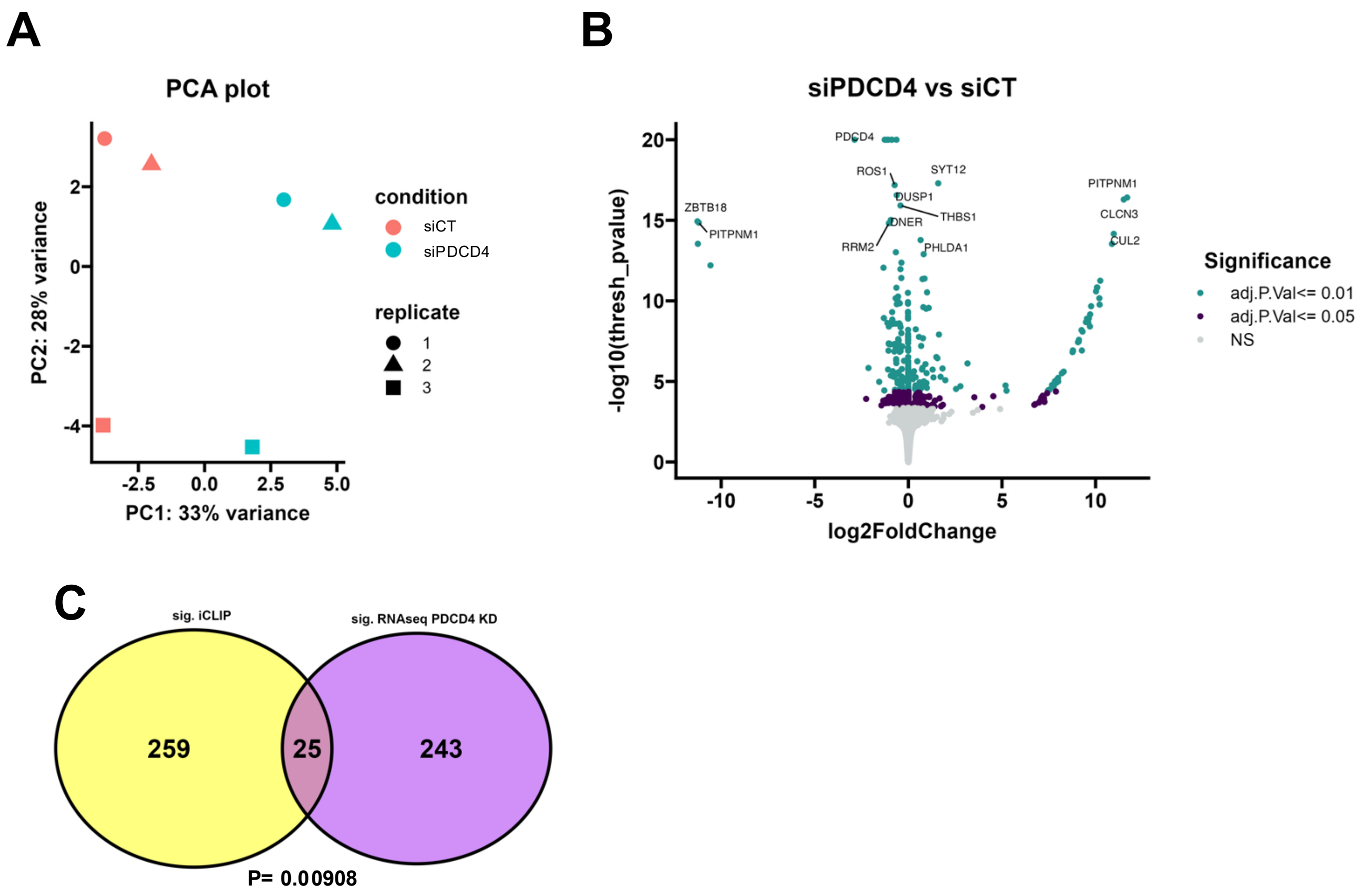

Supplementary Figure 4. A) Principal component analysis (PCA) of siCT and siPDCD4 RNAseq samples. B) Volcano plot representing differential transcripts expression upon PDCD4 silencing; x and y axes represent log2 fold change and –log10 P-value, respectively; in green and red are transcripts with adjusted P-value (BH adjustment) less than 0.05 and 0.01, respectively. C) Venn diagram showing the number of transcripts significantly bound by PDCD4 (PDCD4 vs IgG iCLIP) and the expression of which was significantly (FDR <5%) deregulated upon PDCD4 knockdown. Empirical p-value of the overlap (calculated as the number of times the random overlaps > observed overlaps, divided by n) is shown.

Suppl. Figure 5

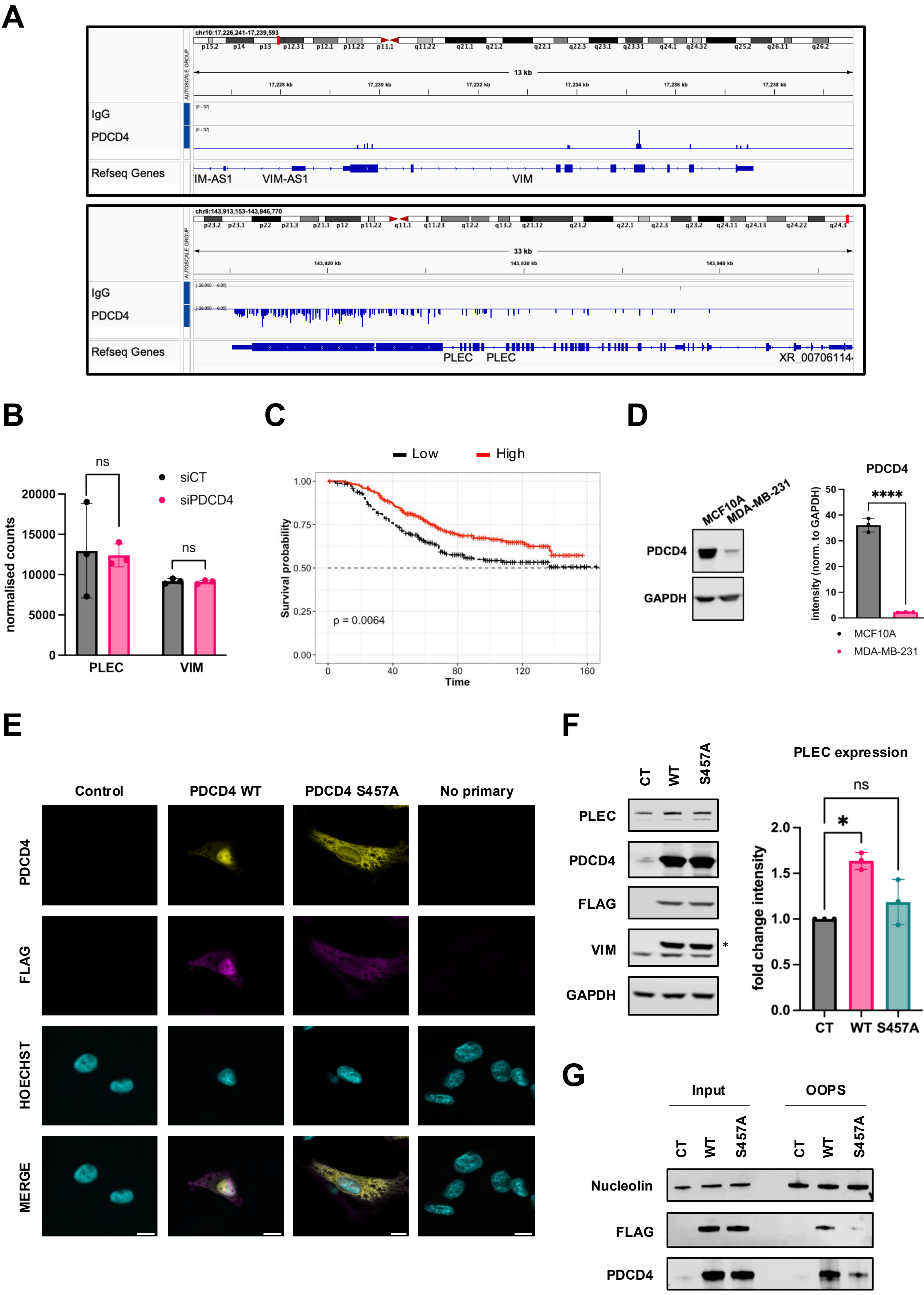

Supplementary figure 5. A) Representation of *PLEC and VIM* loci on IGV (Integrated genome viewer) comparing PDCD4 and IgG iCLIPs. B) Bar chart showing the levels of PLEC and VIM RNAs (expressed as normalised count) in siCT and siPDCD4 cells (unpaired t-test was applied, N=3). C) Survival curves are plotted for breast cancer patients with either low or high expression of PDCD4. Data obtained from GENT2 (http://gent2.appex.kr/gent2/, N=495). D) Left, western blot of whole cell lysates from MCF10A and MDA-MB-231 cells. Right, bar chart comparing the levels of PDCD4 protein in MCF10A and MDA-MB-231 cells (**** = p-value <0.0001, unpaired t-test, N=3). E) Confocal microscopy of MDA-MB-231 overexpressing WT PDCD4 or S457A mutant PDCD4. Cells were stained against PDCD4 (yellow) or FLAG (magenta) and cells nuclei with Hoechst (scale bar, 10 µm). Control cells were untransfected. Images were acquired on Zeiss LS880 confocal microscope. F) Left, representative western blot of whole cell lysates from MDA-MB-231 cells overexpressing WT and mutant S457A PDCD4. The asterisk marks the FLAG band from previous blotting. CT = untransfected. Right, bar chart with densitometry of PLEC protein levels (Kruskal-Wallis statistical test was applied, N=3, * = p-value <0.05). G) Western blotting analysis of input and OOPS samples from MDA-MB-231 cells transfected WT PDCD4 or S457A mutant. Control cells (CT) were untransfected. Blots shown are representative of 3 independent biological experiments.
